# Rosemary Essential Oil Microemulsion for Fungal Keratitis Treatment

**DOI:** 10.1002/cbdv.202502124

**Published:** 2025-10-15

**Authors:** Saulo Ribeiro, Mariana Coelho Moraes, Denise de Oliveira Scoaris, Jovita Eugênia Gazinelli Cruz Madeira, Silvia Ligório Fialho, Carolina Paula de Souza Moreira

**Affiliations:** ^1^ Serviço De Desenvolvimento Tecnológico Farmacêutico, Diretoria de Pesquisa e Desenvolvimento Fundação Ezequiel Dias Belo Horizonte Brazil; ^2^ Serviço De Fitoquímica e Prospecção Farmacêutica, Diretoria de Pesquisa e Desenvolvimento Fundação Ezequiel Dias Belo Horizonte Brazil; ^3^ Serviço De Bioquímica, Instituto Octávio Magalhães Fundação Ezequiel Dias Belo Horizonte Brazil

**Keywords:** drug delivery, keratitis, microemulsion, ocular, *Rosmarinus*

## Abstract

This study investigated the use of *Rosmarinus officinalis* (rosemary) essential oil in a microemulsion (ME) formulation for the treatment of ocular fungal keratitis, a serious infection that can lead to blindness. The ME was characterized in terms of pH, stability, homogeneity, osmolarity, and other physicochemical properties. The Hen's Egg Test‐Chorioallantoic Membrane assay was used to assess ocular irritancy, and antifungal efficacy was evaluated using the minimum inhibitory concentration method. The oil extraction yielded 1.2%, with eucalyptol (37.89%) as the major compound. The formulation showed a suitable pH (6.96), particle size of 11.35 nm, good stability, and was classified as non‐irritant with an irritation score = 1.5. It exhibited strong antifungal activity against *Candida parapsilosis* (96.1%), *C. krusei* (100%), *C. albicans* (99.1%), *Fusarium graminearum* (90.5%), and *Aspergillus parasiticus* (79.5%). The results highlight the potential of rosemary essential oil as a base for developing eye drops for the treatment of fungal keratitis.

## Graphical abstract

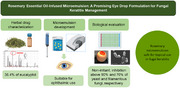

## 1 Introduction

Ocular infection induced by bacteria and fungi without proper treatment is a major cause of preventable visual impairment and blindness [1, 2]. Different types of microorganisms can cause diseases of the human eye. Amongst all the ocular fungal infections, fungal keratitis, endophthalmitis, conjunctivitis, and blepharitis are some of the major clinical concerns. These ocular infections can occur due to numerous factors, such as eye surgeries, ocular trauma, excessive use and/or contamination of ocular products, immunocompromised morbidities, and systemic fungal infections are usually found localized in ocular tissues such as cornea, aqueous and/or vitreous humor, sclera, and the other ocular coats [3].

Mycotic keratitis, a fungal infection with global distribution, if not properly treated, can spread to other parts of the eye, leading to partial loss of vision and, in severe cases, blindness or even total loss of the eyeball [2, 4]. The incidence of corneal keratitis was estimated to be one million cases per year, varying according to the geographic region [5]. Two basic forms of fungal keratitis are recognized: one due to filamentous fungi (especially *Fusarium* and *Aspergillus*), which commonly occurs in tropical and subtropical areas; and another due to yeast or related fungi (in particular *Candida*) [2, 3, 6].

Corneal trauma (primarily with vegetative matter) has been considered the predominant predisposing factor to keratomycosis. However, recently, contact lens usage has been recognized as a significant host risk factor implicated in corneal infections, especially in developed countries [4, 6].

Mycotic keratitis is primarily managed with topical drugs: amphotericin B (AMB), natamycin (NTM), and voriconazole (VCZ) are the topical antifungal drugs with clinical indications, with NTM being the drug of first choice and AMB the best choice for *Aspergillus* and *Candida* keratitis [4, 7]. NTM is epitheliotoxic with prolonged use, leading to ocular irritation, discomfort, and particle retention on the ocular surface, while AMB requires intraocular administration [8]. However, the therapeutic efficacy of current antifungal agents is often limited by important drawbacks, such as the emergence of resistant strains, poor ocular penetration, and, in some cases, systemic toxicity [9]. These limitations highlight the need for alternative or complementary therapeutic approaches, thereby justifying the present study.

The use of medicinal plants has a long history in the treatment of a range of diseases, including infectious diseases, and hundreds of thousands of plant species have been tested for their medicinal properties [10]. Herbal‐formulated medicines and traditional health practices are considered more affordable and accessible to most rural societies than modern drugs [11]. In addition, approximately 39% of the drugs developed since 1980 have been derived from plants and their derivatives [12].

*Rosmarinus officinalis* L., popularly known as rosemary, has promising biological effects, including antimicrobial, antibiofilm, anti‐inflammatory, antioxidant, anti‐ulcer, antiviral, and anticancer. Its antifungal activity was tested against *C. albicans, C. dubliniensis, C*. *glabrata, C. krusei*, and *C. tropicalis*, and it was as effective as nystatin [13]. Essential oils of rosemary have been used as an alternative in the treatment of aspergillosis and have shown positive results in the in vitro models [14, 15]. Rosemary oil, 1,8‐cineole, and α‐pinene disrupt *C. albicans* growth and virulence, showing synergistic antifungal effects and potential as adjunct therapies or antifungal formulations [16].

The core of ocular infection management is the eradication of pathogens, which requires efficient ocular drug delivery to maintain a therapeutic concentration of antimicrobial agents in the infection site. To enhance delivery efficiency and avoid complications of invasive methods, nanomedicine is proposed as a prospective platform to mediate ocular delivery and manage ocular infections [1, 15]. The impenetrability of the corneal epithelium layer associated with short retention time of drugs means that drugs administered as eye drops have reduced corneal permeability and consequently low bioavailability.

Compared to other ocular drug delivery systems, microemulsions (MEs) offer several advantages for topical antifungal application. Their nanometric droplet size ensures optical transparency and enhances corneal permeation, while their thermodynamic stability contributes to longer shelf life and reproducible formulation. In addition, MEs efficiently solubilize lipophilic antifungal agents, such as essential oils, thereby improving their bioavailability and therapeutic potential. However, unlike liposomes or solid lipid nanoparticles, MEs often require higher concentrations of surfactants and co‐surfactants, which may increase the risk of ocular irritation if not carefully optimized. Despite this limitation, their simplicity of preparation, stability, and capacity to enhance local drug delivery make MEs a particularly promising platform for the development of topical antifungal eye drops [17, 18, 19]. Because of that, the aim of this work was to develop an ME with essential oil of rosemary and evaluate its efficacy as an antifungal agent with a view to obtaining eye drops for fungal keratitis.

## 2 Results and Discussion

### 2.1 Essential Oil Extraction and Characterization

The obtained essential oil presented a light‐yellowish color, with a density of 0.89 g/mL and a yield of 1.2% w/w, which is in accordance with previous studies that described a range from 0.5 to 2.5% [20, 21].

Chemical compounds of *R. officinalis* essential oil evaluated by gas chromatography‐mass spectrometry (GC‐MS) are shown in Figure S1 and described in Table 1, with their relative area percentages and linear retention index. The main compounds found in the oil were eucalyptol (1,8‐cineol, 36.4%), camphor (19.9%), α‐pinene (9.7%), isoborneol (7.4%), and L‐α‐terpineol (5.4%). The composition obtained is similar to those previously described by González‐Minero et al. [22], Borges et al. [21], and Ribeiro‐Santos et al. [23], despite the differences in the percentage of each compound, which can be attributed to climatic and cultivation variations, and also according to the vegetative stage.

**TABLE 1:** Chemical composition of Rosemary essential oil.

Peak	Compound	Relative percentage	LRI calc.^[^ ^a^ ^]^	LRI lit.^[^ ^b^ ^]^
1	α‐Pinene	9.65	939	939
2	Camphene	4.41	953	954
3	β‐Pinene	2.04	982	979
4	β‐Myrcene	1.80	994	990
5	o‐Cymene	1.02	1028	1027
6	meta‐Cymene	3.95	1033	1085
7	D‐Limonene	1.21	1037	1029
8	1,8‐Cineole	37.89	1049	1031
9	γ‐Terpinene	0.28	1062	1059
10	Linalool	2.15	1111	1098
11	Camphor	19.86	1164	1146
12	endo‐Borneol	7.26	1181	1169
13	Terpinen‐4‐ol	1.57	1190	1177
14	α‐Terpineol	5.07	1206	1188
15	Bornyl acetate	0.92	1297	1288
16	Caryophyllene	0.74	1425	1408

^[a]^LRI calc: Linear retention index calculated.

^[b]^LRI lit: Linear retention index at literature [21, 22, 23].

Despite the promising application of essential oils, they present limitations in use as medicine because they are volatile compounds susceptible to enzymatic reactions, which can reduce their biological activity and increase their toxicity. Because of that, the formulation in MEs is an important strategy to protect the oil against degradation and increase its bioavailability and efficacy [24]. MEs offer a distinct advantage due to their nanometer‐scale size, which enhances their thermodynamic properties and makes them an effective drug delivery system [25, 26].

### 2.2 Physicochemical Properties and Stability of MEs

The formulation prepared is macroscopically homogeneous and translucent with a pale‐yellow appearance. The physicochemical parameters of the ME containing rosemary oil (MER) were determined. The ME presented a pH value of 6.96 ± 0.07, a value that is included in the physiological range of tears (6.5–7.6), and so it is not expected to cause irritation and discomfort when administered as eye drops [27, 28]. The osmolality of the MER and the blank ME was 495 mOsm/kg. The osmolality of lachrymal fluid typically ranges from 280 to 293 mOsm/kg upon waking. However, when the eyes are open and evaporation occurs, this value can fluctuate between 231 and 446 mOsm/kg. The ocular surface appears to be protected from the potentially harmful effects of the osmolarity ranging from 100 to 640 mOsm/kg [29, 30]. Considering that after instillation of non‐isotonic solutions to the eyes, the original osmolality is restored in 1–2 min, the MER is not supposed to cause discomfort to the eyes, as they are rapidly diluted in tears.

The ME containing the essential oil formulation presented a particle size of 11.35 ± 0.20 nm (polydispersity index [PdI] = 0.101 ± 0.032), and the zeta potential was −7.10 ± 2.37 mV. These results suggest that the formation of MEs, according to the literature, their droplet size varies from 5 to 200 nm [31]. The small size of particles on ophthalmic preparations is desirable as it can increase the comfort during application by reducing a rough feeling, contributing to a higher bioavailability in the eye as small particles may more easily penetrate through the cornea [32, 33]. It is known that high zeta potential values (greater than ±30 mV) can stabilize nanoformulations through electrostatic repulsion and positively charged particles are more effective at enhancing electrostatic interactions with the negatively charged ocular surface [34], however, although the potential of the ME was small and negative, MER proved to be stable during the time of experimentation, maintaining the values of the physicochemical parameters obtained after its preparation.

The value obtained for the viscosity of the ME containing essential oil was 156.65 ± 1.83 cP. The viscosity normally observed for an ophthalmic solution is approximately 20 cP, which ensures good tolerance and does not influence the blinking effect. However, formulations with higher viscosity may increase corneal contact time, as it reduces the drainage rate, allowing an improvement in bioavailability. The viscosity of the developed ME allows sterile filtration and dispensing as eye drops, and despite its higher value when compared with an ophthalmic solution, it may not cause discomfort or blurred vision and can positively increase residence time [35].

Preliminary stability that consisted of centrifugation and ice/thaw cycle tests demonstrated no changes in color, turbidity, precipitation, or phase separation. This indicates optimal accelerated stability of the ME against physical force or temperature changes, which is corroborated by the thermodynamic stability characteristic of this dosage form.

### 2.3 Ocular Safety of Essential Oil MEs

In order to evaluate the safety of the ME for the use as eyedrops, we realized the Hen's Egg Test‐Chorioallantoic Membrane (HET‐CAM) test, which is an alternative assay to the use of animals formerly used by regulatory agencies and that may predict the potential of ocular irritation of the applied formulation through the determination of the parameters of hemorrhage, vascular lysis and coagulation [36, 37]. It uses the CAM of chicken embryonated eggs, which have vascular and inflammatory processes similar to those of rabbit conjunctival tissue [37].

**FIGURE 1:**
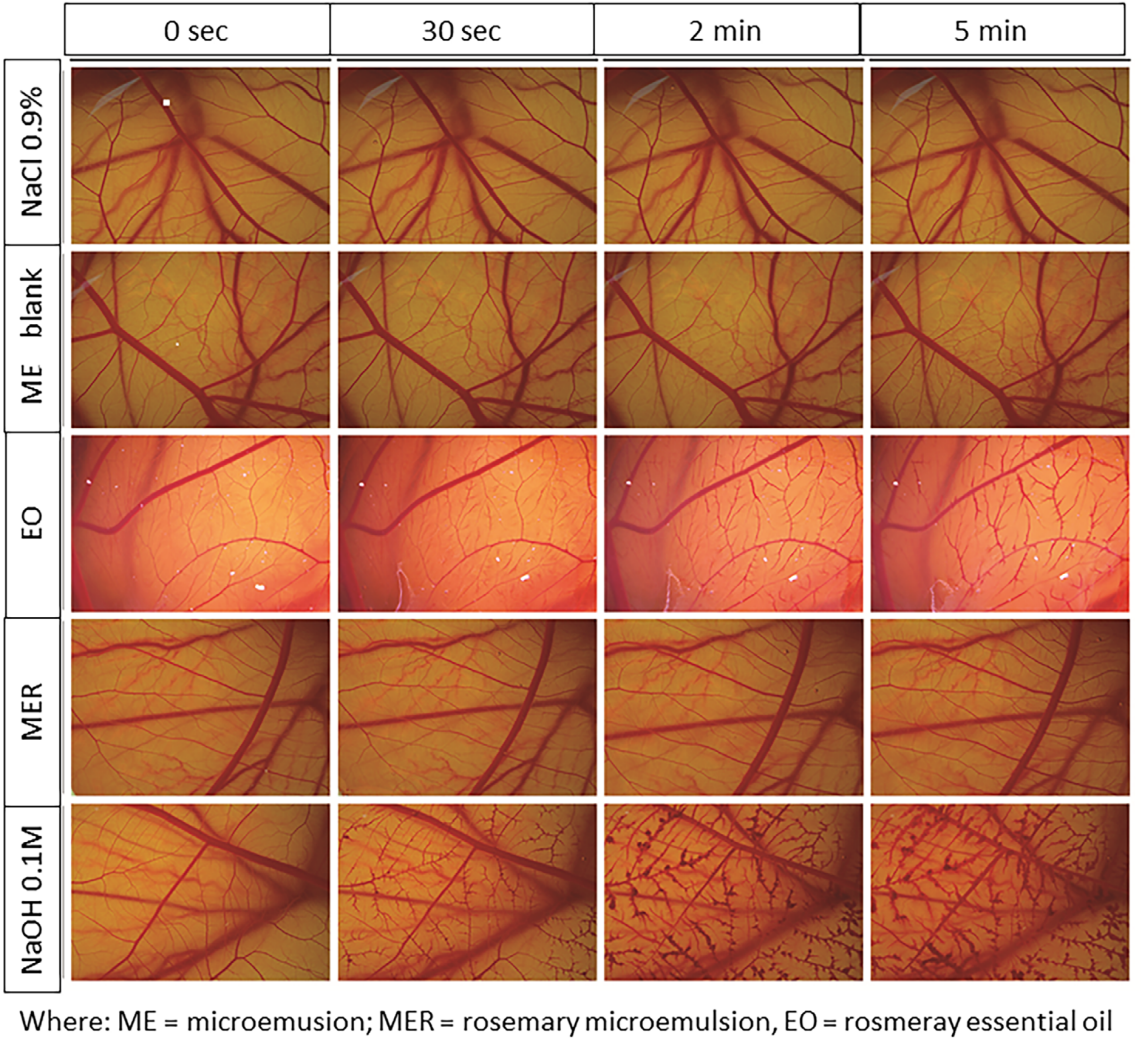
Profile of vascular observations over time to determine irritation potential using the Hen's Egg Test‐Chorioallantoic Membrane (HET‐CAM) method.

The MER and blank formulation were evaluated in the HET‐CAM assay, as well as the essential oil alone. The results were obtained from the averages of sextuplicate, and the tested samples were classified as non‐irritant (irritation score [IS] = 0–0.9), mild irritant (IS = 1–4.9), moderate irritant (IS = 5–8.9), and severe irritant (IS = 9–21). As expected (Figure 1), the application of 0.9% NaCl solution (negative control) did not cause irritation within the 5 min of the test, while NaOH solution (positive control) led to hemorrhage and hyperemia of the vessels, which gradually increased over time. The blank formulation was classified as non‐irritant, as no change in the CAM was observed up to 5 min. The ME containing the essential oil caused mild hyperemia of the CAM vessels at 5 min, and it was classified as non‐irritant to mild irritant, according to the achieved score. Rosemary essential oil alone at the same concentration used in the ME caused hemorrhage to the CAM, showing a high score in the test, and it was classified as a moderate to severe irritant. Considering the results (Table 2), we can say that the ME formulation demonstrated a clear reduction in the potential ocular toxicity of rosemary essential oil, as evidenced in the HET‐CAM assay. This indicates that incorporation into the ME improves the ocular safety profile of the oil. Nevertheless, complementary ex vivo and in vivo studies are needed to validate its therapeutic applicability.

**TABLE 2:** Classification of eye irritant potential according to the French protocol [37].

Sample	IS	Classification
NaCl 0.9%	0.0	Non‐irritant
ME blank**^[a]^**	0.7 ± 0.5	Non‐irritant
EO**^[b]^**	8.7 ± 1.6	Moderate irritant
MER**^[c]^**	1.2 ± 1.0	Mild irritant
NaOH 0.1M	16.7 ± 1.5	Severe irritant

^[a]^ ME = microemulsion, ^[b]^ EO = Rosemary essential oil, ^[c]^ MER = Rosemary microemulsion.

### 2.4 Antifungal Activity of Essential Oil MEs

The antifungal activity of the formulation (MER) was evaluated for the aforementioned species, and reported in Table 3. The minimum inhibitory concentration (MIC) values against the three yeast species ranged from 0.33 mg/mL (against *C. parapsilosis*) to 10.59 mg/mL (against *C. albicans*). Among filamentous fungi, the MIC values against both *F. graminearum* and *A. parasiticus* were 10.59 mg/mL. It was observed that the ME containing essential oil presented antifungal activity, with a percentage of inhibition of the microbial growth above 90% and 70%, respectively, for the yeast and filamentous fungi evaluated. The antifungal activity of the blank ME was also evaluated. Statistical analysis by analysis of variance (ANOVA) revealed a significant difference in antifungal activity between the essential oil formulation and the blank formulation for all tested fungi, except for *A. parasiticus* (*p* < 0.005). Moreover, the MER showed no statistical difference compared with amphotericin B, underscoring its potential as an alternative topical antifungal therapy. By contrast, itraconazole exhibited variable activity across the tested strains, suggesting strain‐dependent sensitivity and further highlighting the consistent performance of the MER. The control with essential oil was not performed.

**TABLE 3:** Minimum inhibitory concentration (MIC) and inhibition percent (IP) of the microemulsion containing rosemary essential oil.

Microorganism	MIC (mg/mL)	IP MER (%)^[a]^	IP blank ME (%)^[b]^	IP Amphotericin B (12.5 µg/mL)	IP Itraconazole (5.0 µg/mL)
*Candida parapsilosis*	0.33	96.1 ± 3.9	8.8 ± 3.9	96.3 ± 3.1	97.5 ± 1.8
*Candida krusei*	5.29	100.0 ± 0.0	44.2 ± 2.5	92.2 ± 10.8	46.3 ± 5.4
*Candida albicans*	10.59	99.1 ± 0.9	65.9 ± 7.9	94.0 ± 4.4	99.8 ± 0.4
*Fusarium graminearum*	10.59	90.5 ± 6.7	0.0 ± 0.0	100.0 ± 0.0	30.0 ± 9.2
*Aspergillus parasiticus*	10.59	79.5 ± 9.0	ND	100.0 ± 0.0	100.0 ± 0.0

^[a]^ MER: rosemary microemulsion. ^[b]^ ME: microemulsion, ND = not determined.

Antimicrobial susceptibility studies have evidenced the great potential of rosemary essential oil in the treatment of infectious diseases caused by fungi. Carbone and collaborators evaluated the in vitro activity of this essential oil against *Candida* species, and MIC (v/v) of 2.0% was determined for *C. albicans* and *C. krusei*, and 0.5% for *C. parapsilosis* [38].

Under the same extraction conditions for the essential oil used in the present study, specifically hydrodistillation, Soltan et al. reported antifungal activity of *R. officinalis* essential oil against *C. albicans*, *C. tropicalis*, and *Aspergillus* sp., by disc diffusion method, with inhibition zones of 16.0 ± 0.5, 13.0 ± 0.0, and 14.0 ± 0.0 mm, respectively. The authors attributed this antifungal activity to the monoterpenes p‐cymene, α‐terpineol, and 4‐terpineol [39].

Additionally, Celiktas et al. demonstrated anticandidal activity of *R. officinalis* essential oil against *C. albicans*, reporting MIC values ranging from 2.5 to 10 mg/mL, similar to those observed in our study [40]. The authors also noted significant variation based on the season of plant collection, with autumn exhibiting the greatest antifungal activity. Interesting to note that the ethyl acetate extract of *R. officinalis* was not able to inhibit the growth of *C. albicans*, highlighting the antifungal potential of the essential oil from this species [41].

In one trial, the MIC of 150 mg/mL was determined against *Fusarium verticilioides* [42]. For the species *Aspergillus niger* and *A. flavus*, the MICs of 625 and 500 µg/mL were established, respectively [43, 44]. The activity against *A. niger* was also recently evaluated, where concentrations of 5% were the most effective [45].

Although some of the MIC values obtained in the antifungal susceptibility assays were higher than those reported in the literature [44], the concentration range with antifungal activity is significantly below the concentration capable of causing ophthalmic irritation, according to analysis in HET‐CAM (22.43 mg/mL). Thus, it is possible to infer that the use of the developed ME is safe, even considering repeated administration throughout the day.

Due to their nanometric size and surfactant properties, MEs also potentiate antimicrobial and anti‐biofilm effects, representing an effective strategy to optimize EO therapeutic use as bioproducts [24]. Karadag and collaborators developed a MER and evaluated its antimicrobial activity against *Candida albicans* [46]. The MIC determined in the assay was 15.6 µg/mL, significantly lower than that obtained with only the essential oil, 62.5 µg/mL. In our study, the MER was also very effective against the tested strains, using low concentrations of essential oil. Compared to the previous study, we obtained inhibition of fungal growth with higher concentrations of essential oil, possibly due to the different components used in the design of both formulations and the variations in the methods of evaluating the antifungal susceptibility.

**FIGURE 2:**
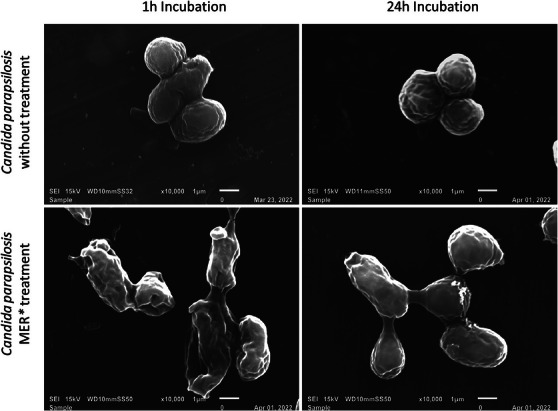
Images taken by scanning electron microscopy of the *Candida parapsilosis* (ATCC 22019) before and after treatment with Rosemary microemulsion (MER) developed.

Corroborating the antifungal activity of ME, SEM images revealed significant morphological alterations in the microorganisms, following treatment with ME. After 1 and 24 h, the control cells predominantly appeared in isolation, forming chains and clusters of interconnected cells. In contrast, the cells treated with ME displayed separation, membrane rupture, and observable fungal debris, indicating cell lysis (Figure 2).

The therapeutic index of the ME was evidenced by irritant concentration determined in the HET‐CAM assay compared with the MIC values obtained against fungal strains, being two times less toxic for *C. albicans*, *F. graminearum*, and *A. parasiticus*, four times less toxic for *C. krusei*, and remarkably 68 times less toxic for *C. parapsilosis*. This substantial reduction in toxicity, combined with the demonstrated antifungal activity, highlights the safety and potential clinical relevance of the ME for ocular use.

## 3 Conclusions

Based on the results of this study, the developed MER demonstrated preliminary in vitro safety for topical ocular application and exhibited potential antifungal activity against clinically relevant fungal keratitis pathogens. Nevertheless, further preclinical animal studies are required to confirm its safety and therapeutic efficacy before any clinical translation can be considered.

## 4 Experimental

### 4.1 Rosemary Essential Oil Extraction and Characterization

For the extraction of rosemary essential oil, dried *R. officinalis* leaves were acquired in Belo Horizonte Central Market (Minas Gerais, Brazil; SISGEN A0420A3) and were hydrodistilled for 3 h in a Clevenger‐type apparatus. The essential oil obtained was collected and dried over anhydrous sodium sulfate and stored in amber glass vials at ‐9°C for use in further chemical and biological studies.

The extraction yield was calculated using the following Equation (1) [20].

(1) $$\mathrm{Yield}\;\left(\%\right)=\left(\frac{\mathrm{Amount}\;\mathrm{of}\;\mathrm{extracted}\;\mathrm{oil}\;\left(g\right)}{\mathrm{Amount}\;\mathrm{of}\;\mathrm{dry}\;\mathrm{vegetal}\;\mathrm{matter}\;\mathrm{mass}\;\left(g\right)}\right)\times \;100\;$$

The essential oil solution (20 µL of the oil in 980 µL of ethyl acetate) was analyzed by GC‐MS using the GCMS‐QP 2010 Ultra system (Shimadzu) with a SH‐RTx‐5MS capillary column (crossbond 5% diphenyl/95% dimethyl polysiloxane, 30 m × 0.25 mm and 25 µm ID, Shimadzu). The initial temperature of the oven was set at 60°C and maintained for 3 min, followed by an increase to 290°C at a rate of 10°C/min, then maintained for 30 min. The injector temperature was set at 220°C, and the helium flow rate was 0.92 mL/min. The ionization voltage was 70 eV and the samples were injected in the split mode (1:5). The mass spectral scanning range used was fixed at 20–500 (*m/z*). A standard mixture of *n*‐Alkanes (C7‐C40 Saturated Alkanes Standard—Supelco) was used to calculate the linear retention index (LRI) of each compound in the sample (Equation (2)). The standard (1 µL of 2%v/v solution in ethyl acetate) was injected into the GC‐MS system, according to the method described above. The identification of compounds was performed by calculating and comparing the similarity index, based on the retention time of each compound, with the National Institute of Standards and Technology (NIST11) library. The calculated LRI values were compared with literature data to confirm compound characterization [47]. The essential oil composition was analyzed on a single representative sample.

(2) $$\mathrm{LRI}=\left(\;\frac{t_{x}\;-\;t_{n}}{t_{n+1\;}-\;t_{n}}\right)+100\;\times \;C_{n}\;$$

where LRI—linear retention index; *t_x_*—retention time of the compound of interest; *t_n_*—retention time of the previous alkane; *t_n_*_+1_ ‐ retention time of the subsequent alkene; *C_n_*—number of carbons from the previous alkane.

### 4.2 Preparation, Characterization, and Stability of MEs

MEs were obtained according to the method previously described [18]. The aqueous phase consisted of phosphate buffer (60%) and propylene glycol (5%), and the oil phase contained polysorbate 80 (30%), isopropyl myristate (2.5%), and rosemary essential oil (2.5%). Blank formulation was also prepared and contained isopropyl myristate at a concentration of 5% instead of the essential oil. For the preparation, the aqueous and oil phases were separately homogenized using an Ultra‐Turrax T‐25X (IKA) set at 8000 rpm for 10 min at 25°C. The aqueous phase was then gently added to the oil phase and homogenized at 8000 rpm for 20 min. The formulations were kept at room temperature until use.

Mean diameter (nm), PdI, and zeta potential (mV) were determined by dynamic light scattering using a Zetasizer Nano ZS (ZEN3600, Malvern Instruments). Prior to analysis, the formulations were diluted with purified water (1:10). The values of pH were determined at 25°C with a NI PHM pH instrument (Nova Instruments). The osmolality was determined using a cryoscopic osmometer (Osmomat 030, Genotec). The samples were previously diluted with phosphate‐buffered saline (1:5). The dynamic viscosity of the ME was measured using a viscometer apparatus equipped with a CP52 spindle model set at 60 rpm (Brookfield RVDVIII Ultra, Brookfield Engineering Laboratory). All measurements were determined in triplicate at 25°C.

Stability of the ME was evaluated by centrifugation and freeze–thaw cycle tests [48]. First, the formulations were centrifuged (Centrifuge SL 702; Solab Científica) at 3000 rpm for 30 min at room temperature. In the freeze–thaw cycle test, the MEs were maintained at 8°C ± 2°C for 48 h and after at room temperature for a further 48 h. This cycle was repeated twice. In both tests, the formulations were observed for any precipitation and/or phase separation.

### 4.3 Irritative Eye Potential by HET‐CAM

The evaluation of the potential ocular irritancy of the developed ME was realized using the CAM of chicken eggs test as French Protocol [36, 37, 49]. Embryonated eggs were incubated at 37 ± 1°C and 60% relative air humidity using an automatic digital incubator (Premium Ecológica, Brazil). The eggs (n = 6) were randomly divided into the following groups: MER, blank ME, and rosemary essential oil alone. NaOH 0.1 M and NaCl 0.9% were used, respectively, as positive and negative controls.

On the eighth day of embryo development, the eggs were gently opened, the CAM was exposed, and 300 µL of each test substance was applied over the CAM. After 30, 120, and 300 s, the irritant effect was evaluated by monitoring the following endpoints: hemorrhage, vascular lysis, and coagulation. The level of irritancy was classified according to the ICCVAM scoring scheme by calculating the IS, which is the sum of numerical time‐dependent scores for the evaluated endpoints [36, 37]. Samples are classified as practically non‐irritating (IS = 0–0.9); mild irritant (IS = 1–4.9); moderate irritant (IS = 5–8.9), and severe irritant (IS = 9–21).

### 4.4 In Vitro Antifungal Activity

Fungal strains used in this study were the yeasts *Candida albicans* ATCC 36802, *C. krusei* ATCC 20295, *C. parapsilosis* ATCC 22019, and the filamentous fungi *Fusarium graminearum* IMI 263189 and *Aspergillus parasiticus* IMI 242695, which were selected by their relevance in clinical fungal keratitis. The fungal strains used in this study were sourced from certified culture collections: yeasts from the American Type Culture Collection (ATCC) and filamentous fungi from the International Mycological Institute (IMI), managed by CABI (Centre for Agriculture and Bioscience International). All strains are classified as biosafety risk level 2 and were therefore handled in biosafety level 2 (BSL‐2) laboratories, adhering to all necessary safety protocols to ensure the protection of both the handler and the environment.

The yeasts were maintained at ‐80°C in GYMP broth (2.0% glucose, 0.5% yeast extract, 1% malt extract, and 0.2% monobasic sodium phosphate). The filamentous fungi, lyophilized and kept at ‐80°C, were rehydrated according to the supplier's instructions and kept in 0.9% saline solution, at 2 to 8 ⁰C, until the tests were performed. Susceptibility tests against yeasts were performed according to CLSI M27‐A3 [50]. The yeasts were grown in Sabouraud Dextrose‐Agar (SDA) plates at 37°C for 18–24 h. After this period, the inocula were adjusted in saline solution using a spectrophotometer at 530 nm to the concentration of 1–5 × 10^6^ CFU/mL, then diluted in RPMI‐1640 buffered with MOPS, at the concentration of 0.5–2.5 × 10^3^ CFU/mL. Serial 2‐fold dilutions of the ME were prepared to obtain final concentrations ranging from 10.59 mg/mL to 330 µg/mL. Volumes of 100 µL of each inoculum were added to the wells containing 100 µL of the ME at different concentrations. Anfotericin B (Pharmédice) at 12.5 µg/mL was used as a positive control. The control of microbial growth was carried out by adding the inoculum to the medium. Sterility of the culture medium was confirmed by its incubation in the assay plate. The ME without the rosemary essential oil incorporation was used as the control of the formulation (blank). The antifungals amphotericin B (12.5 µg/mL) and itraconazole (5.0 µg/mL) were used as positive controls. Assays were performed in 96‐well microplates, in duplicate. The microplates were incubated at 37°C for 24 h. As an indicator of microbial growth, 20 µL of 2,3,5‐triphenyltetrazolium chloride (TTC) (Sigma) at 5 mg/mL was added to each well. The plates were incubated at 37°C for 3 h. Then the TTC was solubilized with 100 µL of sodium lauryl sulfate solution in isopropanol 7 µg/mL and measured in a microplate reader at 485 nm. The result was expressed as the percentage of inhibition of the ME, in comparison with the microbial growth control [51]. The MIC values were determined as the lowest concentration capable of inhibiting ≥90% of the fungal growth [52].

Susceptibility tests against filamentous fungi were performed according to the CLSI M38‐A3 method [53], with modifications. The filamentous fungi were grown in Potato Dextrose‐Agar (PDA) plates at 25°C for 7 days. After this time, *F. graminearum* and *A. parasiticus* were subcultured on potato dextrose agar and malt extract agar (MEA), respectively, at 25°C for 7 days, to stimulate sporulation. Then, the inoculum was adjusted in polysorbate 80 solution at a Neubauer chamber to the concentration of 2 x 10^4^ spores/mL and diluted in RPMI‐1640 buffered with MOPS, at the concentration of 1 x 10^4^ spores/mL. The dilution of the ME and controls of the assay followed the same procedures mentioned for yeasts. Volumes of 50 µL of the inoculum were added to the wells containing 50 µL of the ME at different concentrations. Assays were performed in 96‐well microplates, in duplicate. The microplates were incubated at 25°C for 48 h. The microbial growth was measured in a microplate reader at 620 nm. The result was expressed as performed for yeasts. The MIC values were determined as the lowest concentration capable of inhibiting ≥70% of the fungal growth [52].

### 4.5 Microemulsion Effect in Yeasts Visualized by Scanning Electron Microscopy

The protocol for preparing samples for scanning electron microscopy (SEM) was adapted from Ravensdale et al. [54]. Initially, the microorganisms were washed in 1 mL of phosphate‐buffered saline (PBS) (10^6^ CFU/mL) and centrifuged at 100 rpm for 5 min. The resulting pellets were resuspended in 1 mL of PBS, centrifuged again, and then resuspended in 100 µL of PBS containing 10 µg/mL of the drug. The suspensions were then incubated at 37°C for 1, 6, and 24 h at 200 rpm. Controlled samples were prepared using the same procedure as PBS. Aliquots (15 µL) of each microorganism suspension were deposited onto aluminum stubs, which were then incubated for 30 min at 37°C. Subsequently, the microorganisms were fixed with 2.5% glutaraldehyde for 3 h at room temperature. The stubs were washed with water and then immersed sequentially for 30 min at 37°C in 70%, 90%, and 100% ethanol, respectively. Afterwards, the samples were dried in a desiccator for 24 h at 37°C on silica gel. Finally, the material was metallized with a 3 nm layer of gold to prepare it for SEM imaging.

### 4.6 Statistical Analysis

Statistical analyses were conducted using R‐Studio (version 2025.05.0). Normality of the data series was assessed using the Shapiro‐Wilk test. Parametric data were compared via ANOVA for multiple comparisons, followed by Tukey's post hoc test to identify significant differences. A 95% confidence interval was adopted for all analyses.

## Author Contributions

**Saulo Ribeiro**—formal analysis; **Mariana Coelho Moraes**—formal analysis and original draft; **Denise Oliveira Scoaris**—supervision; **Jovita Eugênia Gazinelli Cruz Madeira**—supervision; **Silvia Ligório Fialho**—funding acquisition and review & editing; **Carolina Paula de Souza Moreira**—conceptualization, data curation, and original draft.

## Conflicts of Interest

The authors declare that they have no known competing financial interests or personal relationships that could have appeared to influence the work reported in this paper.

## Supporting information




**Supporting File 1**: cbdv70573‐sup‐0001‐FigureS1.docx

## Data Availability

The data that support the findings of this study are available from the corresponding author upon reasonable request.
