# Supplementary material for: Rosemary Essential Oil Microemulsion for Fungal Keratitis Treatment
Source: Chem Biodivers. 2025 Oct 15;22(12):e02124. doi: 10.1002/cbdv.202502124 (PMC12715995; doi:10.1002/cbdv.202502124)

**Figure S1.** Chemical profile of the essential oil of *Rosmarinus officinalis* L. by gas chromatography.


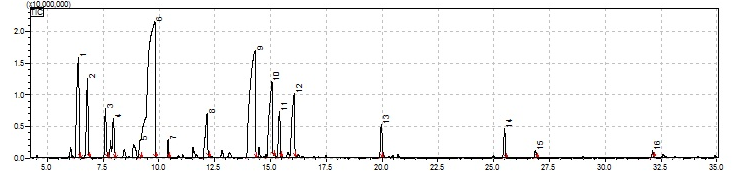

Supplement: Supplementary file 1 — Supporting File 1: cbdv70573‐sup‐0001‐FigureS1.docx [file CBDV-22-e02124-s001.docx]
